# Distribution Patterns of Urban Spontaneous Vegetation Diversity and Their Response to Habitat Heterogeneity: A Case Study of Five Cities in Heilongjiang Province, China

**DOI:** 10.3390/plants13212982

**Published:** 2024-10-25

**Authors:** Haiyan Zhu, Congcong Zhao, Feinuo Li, Peixin Shen, Lisa Liu, Yuandong Hu

**Affiliations:** 1 School of Landscape Architecture, Northeast Forestry University, Harbin 150040, China; 2 School of Resources and Environment, Northeast Agricultural University, Harbin 150030, China; 3 School of Design, The University of Western Australia, 35 Stirling Highway, Perth, WA 6009, Australia; 4 Institute for Interdisciplinary and Innovation Research, Xi’an University of Architecture and Technology, Xi’an 710055, China

**Keywords:** spontaneous vegetation, community structure, plant diversity, environmental characteristic factors, distribution pattern, Heilongjiang Province

## Abstract

Spontaneous vegetation is an important component of urban biodiversity and an excellent agent for exploring the mutual feedback mechanism between urbanization and urban ecosystems. Rapid urbanization has had a significant impact on the composition, structure, and distribution patterns of urban spontaneous vegetation diversity. Studying the diversity distribution patterns and causes of urban plant communities is beneficial for understanding the formation and maintenance mechanisms of plant diversity in specific urban habitats. This study selected five cities in different climate subregions of Heilongjiang Province as research targets and conducted field research using uniform sampling and typical sampling methods. The composition, distribution pattern, and driving factors of spontaneous vegetation were analyzed. The results showed the following: (1) A total of 633 examples of spontaneous vegetation were recorded, belonging to 93 families and 341 genera, mainly consisting of herbaceous plants and native plants. (2) The diversity index and similarity index of spontaneous vegetation in gravel-type abandoned land habitats are higher than those in other habitat types, while the diversity index of spontaneous vegetation in trees and shrubs is lower, and there is no significant difference in regards to different habitats. (3) Urban population density is a key factor affecting the diversity of native plants, while woody plant coverage, patch area, and landscape trait index are key factors affecting non-native plants. (4) The results of canonical correspondence analysis (CCA) showed that the total explanatory power of environmental characteristic factors in regards to the distribution pattern of spontaneous vegetation was 7.5%. The closest distance between adjacent patches, the coverage of woody plants in patches, the distance from the city edge, the patch area, and the surface impermeability of the buffer zones were key factors affecting the distribution of dominant species in spontaneous vegetation communities. The research results will provide an important reference for the conservation of urban biodiversity and the construction of low-maintenance urban green space plant landscapes in Heilongjiang Province.

## 1. Introduction

The rapid economic development and urbanization process has brought significant economic benefits to people, but have also led to a reduction in habitats suitable for natural vegetation, triggering a series of issues, such as a decline in biodiversity, which seriously threaten the sustainable development of both society and cities [1,2]. As an important component of urban biodiversity and as primary producers in urban green spaces, urban vegetation not only provides many economic and social benefits, such as health and aesthetics for humans [3,4], but also plays a key role in maintaining the health of the urban ecological environment, regulating climate and providing biological habitats [5]. In recently years, people have realized that the standardized construction of urban green spaces and their subsequent high-intensity maintenance have led to urban landscape homogenization and difficulties in maintaining biodiversity levels. Therefore, the creation of urban plant landscapes has shifted from primarily focusing on aesthetic benefits to aiming toward comprehensive ecological, social and economic benefits [6]. Compared to neatly and brilliantly cultivated plants that require high maintenance and management costs, spontaneous vegetation offers greater vitality, allowing it to not only exist in urban green spaces, but also to grow on various hard surfaces such as walls, pavement gaps, roofs, etc. [7]. Because of its self-propagation abilities, minimal maintenance and management needs, and adaptability to the local environment, spontaneous vegetation shows great potential to improve the ecosystem services of urban green spaces and construct low-maintenance urban landscapes. It is also an important plant material for building sustainable and low-maintenance urban landscapes [8,9]. In order to help spontaneous vegetation fully exert its ecological and landscape functions, its utilization and protection are key elements in the research regarding spontaneous vegetation in the process of urban development.

Research on urban spontaneous vegetation has a history of over 20 years in countries such as the Czech Republic, the United Kingdom, etc. [6,10,11,12], but in China, in recent years, scholars have begun to focus on the study of urban spontaneous vegetation at the urban, patch, and micro-habitat scale. The research areas are mainly located in the southern temperate zone and subtropical cities of China, such as Kunming, Beijing, Chongqing, Shanghai, Hangzhou, and Nanjing [13,14,15,16,17,18,19,20,21]; however, there are few studies of spontaneous vegetation in the cold regions of northeastern China, except for Harbin. There is a significant correlation between the abundance of spontaneous vegetation in urban environments and heterogeneous habitats [22], but how habitat heterogeneity affects the distribution and maintenance mechanisms of urban spontaneous vegetation diversity is not yet clear. Therefore, this study focuses on the built-up areas of five cities in Heilongjiang Province, located in the cold regions of northeastern China. This study analyzes the resources and composition characteristics of the spontaneous vegetation species of these cities based on field investigations, explores the distribution pattern and causes of spontaneous vegetation diversity, and reveals its formation and maintenance mechanisms in cold regions.

## 2. Results

### 2.1. Composition of Spontaneous Vegetation

**Figure 1:**
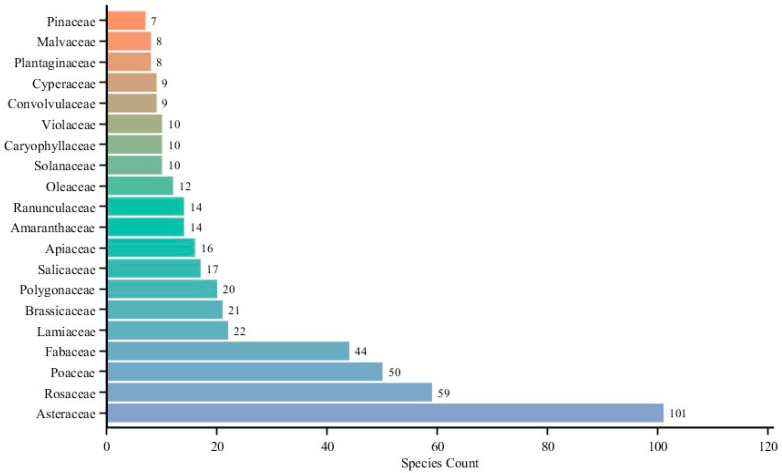
Families with the top 20 species richness.

**Figure 2:**
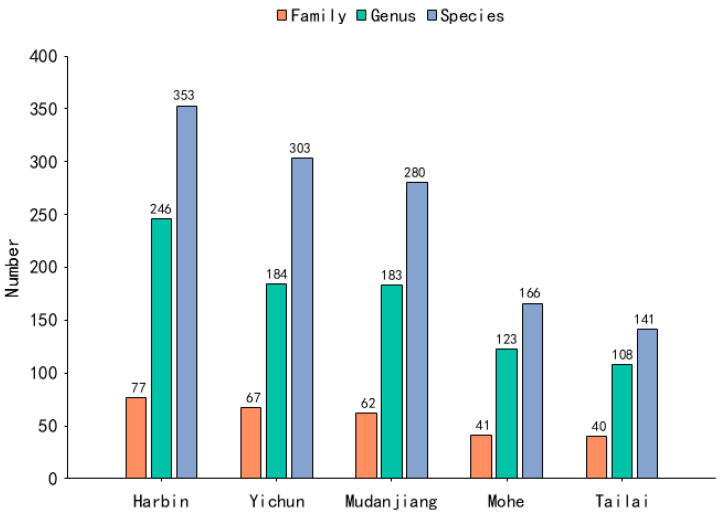
Characteristics of family, genus, and species composition in each city.

A total of 633 examples of spontaneous vegetation belonging to 93 families and 341 genera were recorded in five cities in Heilongjiang Province, and Compositae, Rosaceae, Gramineae, and Fabaceae were the dominant families, with species numbers of 101, 59, 50, and 44, respectively (Figure 1). There are significant differences in the number of examples of spontaneous vegetation in different cities (Figure 2).

From the perspective of life forms composition (Table 1), there are 479 species (75.7%) of herbaceous plants, 71 species (11.2%) of trees, 65 species (10.3%) of shrubs, 15 species (2.4%) of vines, and 3 species (0.5%) of ferns. Herbaceous plants have the highest proportion (84.3%~76.1%), followed by trees (12.9%~8.4%) and shrubs (10.3%~6%) in different cities, and ferns (0.5%~0) and vines (3.7%~1.0%) display relatively low proportions in all cities. Different from other cities, the proportion of perennial herbs in Mohe is significantly higher than that of one- or two-year-old herbs, and the number of shrub life-type species (6%) in Mohe is significantly lower than that in other cities.

From the perspective of species origin (Table 2), among the 633 examples of spontaneous vegetation recorded in the five cities, there are 429 native plants (67.8%) and 204 non-native plants (32.2%). Among the non-native plants, there are 63 invasive plants, accounting for 31.0% of the non-native plant composition. The proportion of native plants in Harbin, Mudanjiang, and Tailai is not significantly different, ranging from 63.2% to 68.8%, while the proportion in Mohe is the highest, reaching 76.5%. The proportion of invasive plants in all cities ranges from 10.6% to 15.0%.

It can be seen that a total of 94 families of spontaneous vegetation were recorded in this study, which can be divided into 14 geographical distribution types (including subtypes). The world’s widely distributed spontaneous vegetation consists of 38 families, accounting for 40.4% of the total number of families. From the perspective of different cities, Tailai boasts the highest tropical geographic component, reaching 61.9%, followed by Mudanjiang (54.3%) and Harbin (52.3%). Different with the three cities, the origin of the spontaneous vegetation families in Yichun and Mohe is mainly composed of temperate elements (Table 3).

### 2.2. Distribution Pattern of Urban Spontaneous Vegetation Diversity

#### 2.2.1. Community Types and Distribution Characteristics of Urban Spontaneous Vegetation in Different Urban Habitat Types

**Figure 3:**
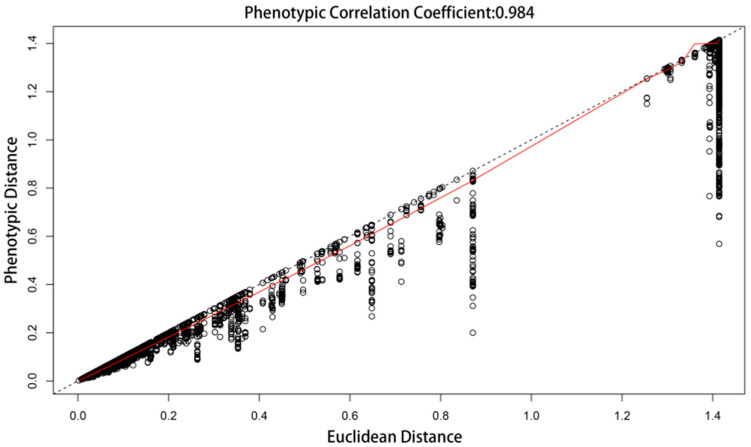
Phenotypic correlation of cluster analysis results with Euclidean distances.

**Figure 4:**
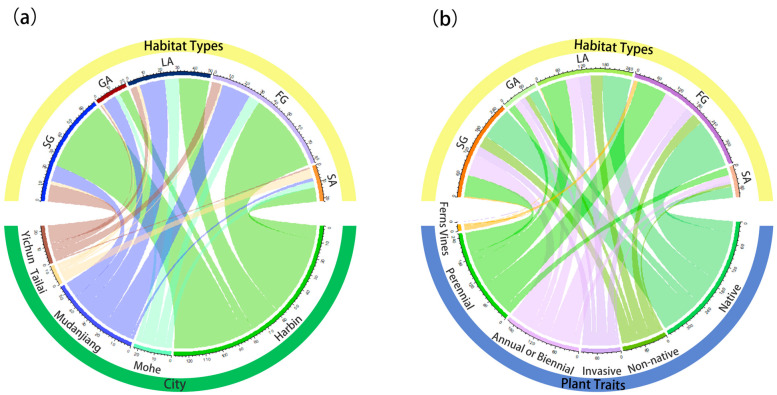
(**a**) Chord diagram illustrating the relationship between cities, habitats, and dominant community types of native plants. (**b**) Chord diagram illustrating the relationship between habitats, plant traits, and dominant community types of native plants.

The research results indicate that the optimal number of groups for spontaneous vegetation communities is 104 species, with a cophenetic correlation coefficient of 0.984 (Figure 3). In different urban habitat types, the number of dominant community types of spontaneous vegetation, from high to low, is FG > SG > LA > SA > GA. Harbin, Mudanjiang, Yichun, and Tailai all display these five habitat types, while Mohe lacks the SG habitat type (Figure 4a). The proportion of dominant species in native spontaneous vegetation communities is greater than that of non-native plants, and most non-native plants are invasive plants. Non-native plants in GA habitat types are all invasive plants. In terms of lifestyle, the dominant species of spontaneous vegetation communities in the five cities are mainly perennial herbs, while fern communities have only appeared in FG habitat types (Figure 4b).

#### 2.2.2. Distribution Patterns of Spontaneous Vegetation Community Diversity in Different Urban Habitat Types

**Figure 5:**
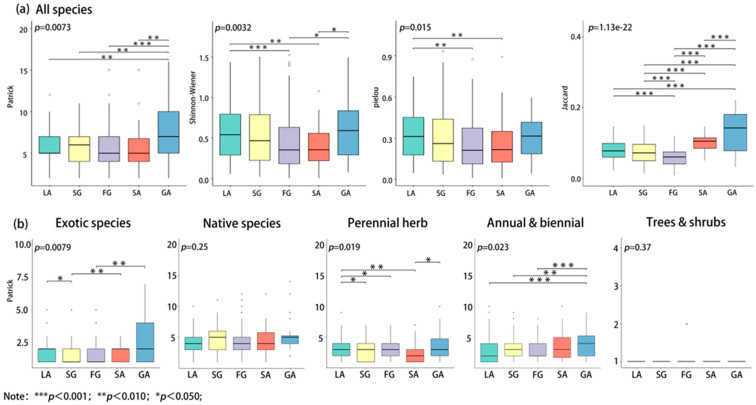
(**a**) Diversity, evenness, and similarity indices of native plants in different habitat types; (**b**) diversity indices of native plants with different plant traits in various habitat types.

The diversity index of spontaneous vegetation shows different distribution characteristics in different habitat types. The Patrick richness index and the Shannon–Wiener diversity index of spontaneous vegetation are the highest in the GA habitat type, while the Pielou evenness index dominates in the LA habitat type and shows significant differences among different habitat types (*p* < 0.05). From a similarity perspective, there are more shared species in the GA habitat, while there are significant differences in regards to species composition in the FG habitat type (Figure 5a). Meanwhile, the species composition of the GA and SA habitat types is relatively similar, whereas the species composition of the FG habitat differs significantly from that of the other habitats (Figure S1). There are significant differences (*p* < 0.05) among non-native spontaneous vegetation types in different habitats (*p* < 0.05), while the differences among native plants are not significant (*p* > 0.05), but the species richness index of native plants is generally higher. The species richness index of perennial herbaceous plants showed significant differences in different habitats (*p* < 0.05), with the lowest value occurring in the SA habitat type. The diversity index of trees and shrubs is relatively low, and the differences in different habitat types are not significant (*p* > 0.05) (Figure 5b).

### 2.3. The Impact of Different Environmental Characteristic Factors on the Distribution Patterns of Spontaneous Vegetation Diversity

**Figure 6:**
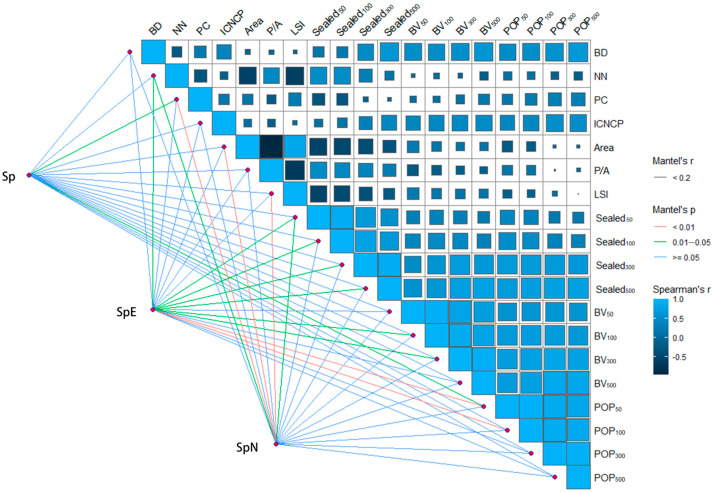
Correlation between native plant richness and environmental factors. Area: patch area; BD: average distance from patch to urban edge; P/A: perimeter–area ratio of patches; LSI: patch landscape shape index; NN: distance between neighboring patches; PC: percentage of woody cover in patches; ICNCP: relative richness of cultivated plants in patches; BV_50/100/300/500_: building volume within 50/100/300/500 m buffer of patches; Sealed_50/100/300/500_: impervious surface within 50/100/300/500 m buffer of patches; POP_50/100/300/500_: population density within 50/100/300/500 m buffer of patches.

There is a significant positive correlation between the average distance from the patch to the urban edge and the impervious surface ratio in the buffer zones of the patches, building volume and population density in the buffer zones of the patches, but the value of Mantel’s r did not show a strong correlation because it contains a significant amount of sporadic components. The richness of spontaneous vegetation, native spontaneous vegetation, and foreign spontaneous vegetation is not highly correlated with the BD, the relative richness of cultivated plants in patches, and the perimeter–area ratio of patches. However, the diversity of native spontaneous vegetation is significantly correlated with population density within the 50/100 m buffer of patches (*p* < 0.01), while the richness of non-native spontaneous vegetation is significantly correlated with the percentage of woody cover in patches, patch area, and patch landscape shape index (*p* < 0.01) (Figure 6).

**Figure 7:**
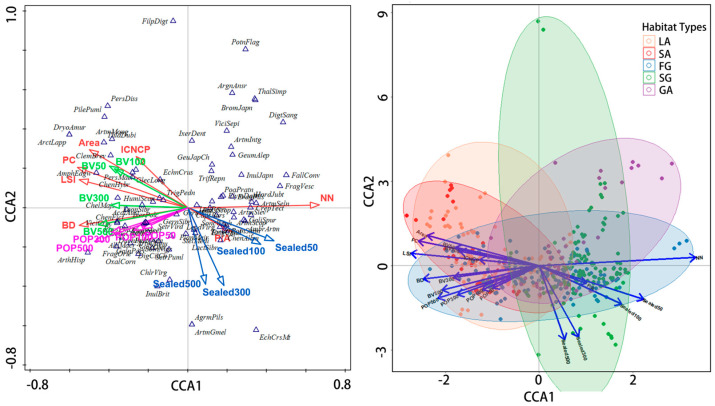
CCA ordination diagram for environmental factors, dominant community species, and quadrats; the plant names are abbreviated here, according to the custom settings in the Canoco 5 software.

The results of the exploratory analysis, based on a canonical correspondence analysis (CCA), indicate that the cumulative variance explained by the first three axes of the fitted data is 31.7%, while the first three axes of the response data explain 2.4% of the variance (Table S1), with a total inertia of 46.381. The variable distance between the neighboring patches, PC, BD, area, and sealed surface show a relatively high explanatory power and a significant contribution to the total variation, with strong statistical significance (*p* = 0.001) (Figure 7) (Table S2), indicating that these variables are the key drivers influencing the distribution of dominant species in spontaneous vegetation communities (Table S3). From the distribution characteristics of dominant species in spontaneous vegetation communities, plants such as *Crepis tectorum* and *Artemisia scoparia* tend to be more distributed in areas with greater NN and stronger sealed surface, and plants such as *Clematis brevicaudat* and *Amphicarpaea edgeworthii* are more affected by area, PC, and LSI, indicating that they are more suitable for areas with larger patch areas, higher vegetation coverage, and more complex landscape shapes. Plants such as *Duchesnea indica*, *Vicia cracca*, and *Chenopodium ficifolium* are more adapted to areas with higher levels of BD, BV, and POP. The correlation between the above variables and species composition varies significantly in different habitat types. POP, BV, LSI, PC, area, and ICNCP are key driving factors affecting the distribution of spontaneous vegetation diversity in LA and SA habitats, and NN, sealed surface, and P/A are key driving factors affecting the distribution of spontaneous vegetation diversity in SG and GA habitats. In the FG habitat, only NN has a relatively low impact on the diversity composition of its species, with POP_50/100/300/500_ as its key driving factor (Figure 7).

## 3. Discussion

### 3.1. The Main Factors Influencing the Diversity and Composition of Urban Spontaneous Vegetation

Compared with the survey results for spontaneous vegetation in nine cities in Yunnan Province [14], which are located in the global biodiversity hotspot area, the number of examples of spontaneous vegetation recorded in this study reached 62.3%, indicating that spontaneous vegetation is widely present in various urban habitat types subject to severe human interference and have important research value and ecological significance. The ranking of the number of spontaneous vegetation community categories in different urban habitat types is similar to those found in the research results of Tian Zhihui et al. in Shanghai [17], indicating that habitat type is a key driving factor affecting the diversity composition of spontaneous vegetation. In different regions, habitat type shows a consistent ranking regarding plant community numbers. The research results show that the families with the highest number of examples of spontaneous vegetation are Asteraceae, Rosaceae, Poaceae, and Fabaceae, similar to the findings of other related research [14,19].

The spontaneous vegetation in urban areas of Heilongjiang Province comprises mainly herbaceous plants, which is similar to the research results for Harbin, Chongqing, Nanjing, Wuhan, and other locations in China [20,23,24,25,26]. Due to their short life cycle, high plasticity, and strong stress resistance, herbaceous plants can quickly respond to highly heterogeneous urban habitats and can colonize in environments of high-intensity and high-frequency human disturbance [26,27]. Among the five cities in Heilongjiang Province, Mohe City, located at the northernmost and coldest point, has a significantly higher proportion of perennial herbaceous plants than one- or two-year herbaceous plants, and the proportion of shrubs is the lowest. This may be related to the ecological and physiological characteristics of perennial herbaceous plants, which boast a stronger survival ability under extreme climate conditions, such as low temperatures [28]. It is difficult for most shrubs to carry out normal physiological activities in cold regions, with adverse effects on their survival [29]. Ferns are considered as indicator plants of forest environmental changes [30], as they only appear in urban forest crevice habitats marked by minimal human interference, weak light, and moist soil.

Overall, the proportion of native plants is significantly higher, which is similar to the survey results for spontaneous vegetation in cities such as Yunnan, Shanghai, and Zhengzhou [14,17,31]. Compared to other cities in Heilongjiang Province, Mohe City has the highest proportion of native plants. This is mainly because native plants can adapt to the local environment over time in the face of extreme weather, such as severe cold, and possess better survival and growth capabilities [28]. This study found that a considerable proportion of the dominant species in the spontaneous vegetation community are non-native plants, many of which are invasive species. This result indirectly indicates that non-native spontaneous vegetation carries huge invasion debts and exhibits a higher probability of becoming invasive in cities [32]. From the perspective of geographical composition, spontaneous vegetation of Tailai, Mudanjiang, and Harbin, located in temperate climate zones, is mainly characterized by tropical geographical components. This may be due to global warming and the urban heat island effect, which have led to an increase in local air temperature in cities, allowing spontaneous vegetation with tropical components to colonize and further affect the distribution of urban plant diversity to varying degrees [33].

The Patrick richness index and the Shannon–Wiener diversity index of spontaneous vegetation in the GA habitat both exhibited the highest values, which may be due to its rich diversity of terrain and substrates, forming a rich variety of micro-habitat types that can better support the diversity of plant communities compared to the conditions displayed by other habitat types. At the same time, the Jaccard similarity index of GA habitats also showed a high level, which may be due to the consistent characteristics of this type of habitat, supporting the combination of similar trait species, thereby improving the similarity of community species composition between different cities, plots, or patches [34]. Additionally, the high species similarity between the GA and SA habitats is primarily attributed to the significant environmental stress in both types of abandoned land habitats, which favor the growth of plant species with strong adaptability and high tolerance. The Pielou evenness index of spontaneous vegetation in the LA habitat type displays relatively high levels, which may be related to the intensity of lawn management and weed control measures. Regular maintenance limits the spread of certain populations, usually resulting in a more uniform distribution of patch species. The significant differences in the composition of spontaneous vegetation in FG habitats, and their distinctiveness from the species composition of other habitats, may be related to the richer and more complex forest layer structure and the micro-environment of this habitat type, which promotes the localization and specialization of species diversity. In addition, the species richness index of native plants is significantly higher than that of non-native plants, indicating that native plants are more adaptable to local environmental conditions and can better adjust to the biodiversity of local cities than can non-native plants [35] The species richness index of perennial herbaceous plants in SA habitat types is relatively low, which may be mainly related to the limitations of urban soil characteristics or other environmental pressures. The specific reasons for this result require further in-depth research. The richness index of woody spontaneous vegetation is relatively low, and the difference is not significant, which is consistent with the results of previous studies [15]. Most woody spontaneous vegetation grows from the seeds or sprouts of cultivated plants, and these seedlings are usually shorter and easier to preserve during weed control and other maintenance processes. However, due to limitations regarding growth conditions, such as the availability of light and nutrients, most of the current year’s woody seedlings experience difficulty surviving without deliberate preservation and conservation efforts [15].

### 3.2. Key Driving Factors for the Distribution Patterns of Spontaneous Vegetation Diversity in Cities

The research results indicate that as the distance from the patch to the city edge increases, the proportion of the impermeable pavement area in the patch buffer zone increases, the building volume increases, and the population density increases, which means that the closer the distance to the city center, the higher the degree of urbanization in the region. There is a significant correlation between the diversity of native plants and the population density within 50/100 m of the patch buffer zone, indicating that human activity interference and its degree in nearby areas are key driving factors affecting the diversity of native plants. Previous studies have shown that human intervention has a dual effect in local plant conservation strategies, potentially leading to species pressure and increasing species richness through conservation management measures [36]. For non-native plants, larger patches usually provide more resources and ecological niches, allowing for more opportunities for colonization and dispersal. At the same time, it also indicates that within a certain urbanized area, larger green spaces can accommodate a richer variety of spontaneous vegetation. Higher density forests or tree canopies often limit non-native plants that require sufficient light for rapid growth or dispersal, especially for invasive species. At the same time, native species may more effectively utilize water and soil conditions in the forest, and strong competitive advantages can help suppress non-native plants with higher resource demands [37,38]. The richness of non-native plants is significantly correlated with LSI, mainly because complex-shaped patches can provide more significant edge effects, diverse habitats, more opportunities for introduction and dissemination, and highly heterogeneous habitats for resource utilization.

The CCA analysis results suggest that the overall explanatory power of urban environmental characteristic factors for the distribution of dominant species in the spontaneous vegetation communities in Heilongjiang Province is relatively low, but they may still have some explanatory power in regards to specific spatial scales and habitat types. This result indirectly reflects the complexity of the distribution pattern of urban biodiversity, and multivariate environmental characteristic factors often only provide limited explanatory power in complex urban ecological environments. The research results indicate that NN, PC, BD, area, and sealed surface are the main driving factors affecting the distribution of dominant species in spontaneous vegetation communities. Among them, plants such as *Crepis tectorum* and *Artemisia scoparia* tend to be distributed in areas with greater NN and higher sealed surface, indicating that these species can adapt to or may prefer urban environments with stronger species dispersal resistance [14]. Plants such as *Clematis brevicaudata* and *Amphicarpaea edgeworthii* tend to be distributed in areas with larger green spaces, higher vegetation coverage, and more complex landscape shapes because rich resources and diverse habitats help them to inhabit these areas [39]. Plants such as *Duchesnea indica*, *Vicia cracca*, and *Chenopodium ficifolium* display strong adaptability to human activity interference and also show distribution advantages in high population density and large building volume habitats.

The correlation between environmental variables in different habitat types shows significant differences, indicating that targeted protection and optimization measures are also necessary in different urban habitats. Urban biodiversity planning and design should fully consider specific influencing factors from different fields, such as soil conditions and their various variables, along with light intensity, as well as the key factors mentioned above, to effectively promote the conservation and maintenance of urban plant diversity. Specific suggestions include the following: (1) The distance between green patches and the proportion of impermeable surfaces should be reasonably planned to reduce resource competition pressure and the intensity or frequency of human interference activities. (2) The area of green patches and the coverage of woody plants can be increased to enhance the stability and diversity of the ecosystem by planting trees and implementing greening measures. (3) Complex and diverse green landscape shapes should be maintained as much as possible, and rich and diverse habitat types should be created. (4) Efforts should be made to protect and restore remaining natural patches on the outskirts of cities to reduce the negative impact of urbanization on them and to enhance the connectivity of ecological networks through green belts and ecological corridors. (5) Fragmentation effects should be minimized as much as possible during the process of designing small green patches.

### 3.3. Recommendations for Managing and Maintaining Spontaneous Vegetation in Low-Maintenance Urban Landscape Construction

To add wildness to the urban landscape and reduce the maintenance costs of urban green spaces, spontaneous vegetation should be reasonably preserved and utilized [21]. In the future planning and design of urban green space plant landscapes, flexible maintenance and management strategies should be adopted based on different habitat types and vegetation group structures. Based on the results of this study, the following suggestions are proposed for the maintenance and management measures of different urban habitat types, and suitable species are recommended for the creation of low-maintenance plant landscapes (Table 4).

(1) Artificial turf habitat: Due to strong human interference and the absence of other vegetation covers, it is recommended to prioritize selecting herbaceous plants with strong stress resistance, such as *Potentilla chinensis*, *Medicago lupulina*, *Plantago asiatica*, etc., to maintain the proportion of native plants and stress tolerant grass species and improve the ecological stability of the turf. It is also recommended to reduce the frequency of pruning, irrigation, and fertilization, allowing spontaneous vegetation to naturally colonize and retain those that display better landscape effects. In sparse areas of the lawn, spontaneous vegetation can be introduced appropriately, which can not only gradually restore degraded habitats, but also achieve the rewilding of artificial lawn landscapes and maintain biodiversity [40].

(2) Forest crevice habitat: Most of these habitats are covered by trees or shrubs. In the process of plant landscape design, it is recommended to prioritize shade tolerant plants such as *Impatiens noli-tangere*, *Athyrium brevifrons*, *Chelidonium majus*, etc. It is also recommended to employ minimal intervention measures to reasonably control the dominant invasive plant population, prevent vicious competition with native plants, and enhance the self-diffusion and renewability of native plants. It is recommended to replant shade tolerant herbs and shrubs in areas with sparse vegetation to increase vegetation coverage. In addition, while preserving existing native plants, a strategy that combines natural succession with artificial replanting is adopted to form a stable plant community landscape.

(3) Grassland crevice habitat: The water conditions in such habitats may fluctuate continuously due to the uneven distribution of vegetation and the complexity of ecological processes. It is recommended to choose drought tolerant native plants with strong tolerance to shade and adaptability for supplementation, such as *Viola collina*, *Lespedeza bicolor*, *Gueldenstaedtia verna*, etc. It is also recommended to minimize artificial intervention as much as possible to increase the diversity and ecological resilience of self-growing plants [41]. In areas with sparse vegetation or a lack of native plants, moderate artificial replanting should be carried out to enhance species diversity, providing only the necessary water and nutrients in the early stages of vegetation establishment and gradually reducing management inputs thereafter.

(4) Gravel-type abandoned land and barren soil abandoned land habitat: It is recommended to moderately introduce adaptable native pioneer plants, such as *Digitaria sanguinalis*, *Setaria viridis*, *Eragrostis pilosa*, etc. These plants not only have the ability to rapidly spread, but can also stably inhabit complex urban ecological environments and contribute to soil remediation [6,42]. Implementing this process not only helps with initial vegetation restoration, but also forms a biological matrix, laying the foundation for subsequent benign succession directions. To ensure the success of ecological restoration, it is recommended to regularly monitor changes in vegetation community structure and take effective measures to strictly control invasive plants.

## 4. Materials and Methods

### 4.1. Study Area

**Figure 8:**
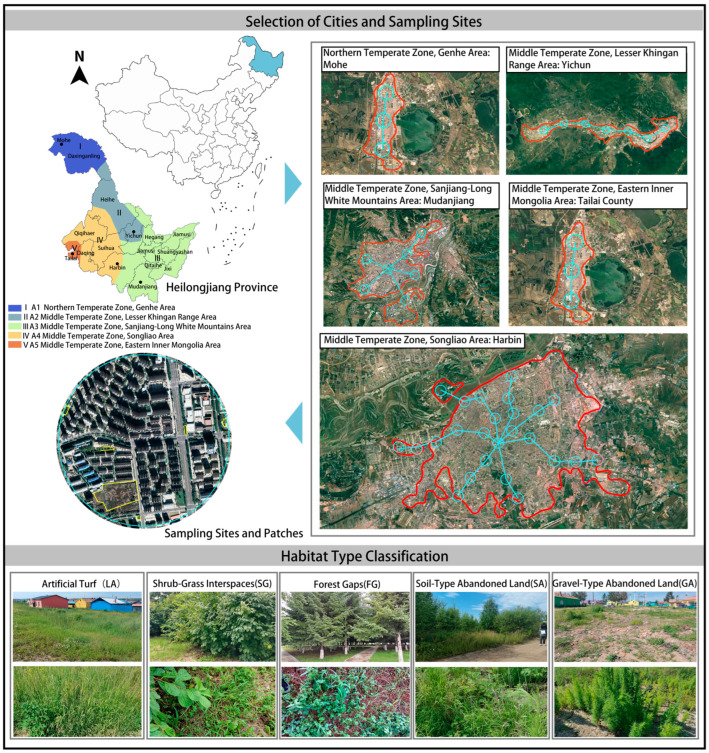
Schematic diagram of survey sample points and habitat types in the study area.

Heilongjiang Province (43°26′–53°33′ N, 121°11′–135°05′ E) is located in the northeastern region of China. It belongs to the cold temperate and temperate continental monsoon climate, with warm and rainy summers and long and cold winters, along with significant climate differences between cities [43,44]. According to temperature and dryness indicators, Heilongjiang Province is divided into five climate subregions, with one city selected from each subregion as the research area. Mohe, located in the Northern Temperate Zone (Genhe Area), is characterized by extremely cold and long winters, short mild summers, and low precipitation. Yichun, in the Middle Temperate Zone (Lesser Khingan Range Area), has a cool climate, with concentrated summer rainfall and rich vegetation. Mudanjiang, also in the Middle Temperate Zone (Sanjiang-Long White Mountains Area), shares a similar climate with Harbin but experiences slightly colder winters due to its proximity to mountainous areas, along with higher precipitation levels. Harbin, situated in the Middle Temperate Zone (Songliao Area), has a temperate continental climate with warm, humid summers and cold, dry winters. Finally, Tailai, located in the Middle Temperate Zone (Eastern Inner Mongolia Area), is characterized by a relatively dry climate, with hot summers, cold winters, and less precipitation (Figure 8).

### 4.2. Spontaneous Vegetation Survey Methods

Based on the geometric shape of the built-up areas in each city, starting from the city center, “—” - or “✱” -shaped transects are laid out along the urbanization gradient. A circle with a radius of 500 m is set every 2 km on each transect as the sampling area, and five different types of green patches are selected within each circle as sampling areas for vegetation investigation [14]. The vegetation survey was conducted between June and September 2023. the typical communities within the patches were surveyed and documented using the phytosociological method of the Zurich–Montpellier School [45]. Based on previous studies and study area characteristics [17,18,19,46,47], the sample plots were divided into five habitat types: artificial turf (LA), shrub–grass interspaces (SG), forest gap (FG), soil-type abandoned land (SA), and gravel-type abandoned land (GA) (Figure 8).

### 4.3. Data Analysis Method

#### 4.3.1. Calculation of Diversity Index

(1) Species dominance

The formula for calculating the relative dominance of species is as follows [48]:

(1) $$D=\left(IV/\Sigma IV\right)*100\%,$$

(2) IV=H∗C,

where *D* is the relative dominance of the species in the community; *IV* is an important value; *H* is the maximum height of the species; *C* is the species coverage.

(2) *α* diversity

The formula for calculating Patrick’s richness index is as follows [49]:

(3) R=S,

The formula for calculating the Shannon–Wiener diversity index is as follows [50]:

(4) $$H=-\sum_{i=1}^{P}P_{i}\ln P_{i},$$

The calculation formula for the Pielou uniformity index is as follows [51]:

(5) $$J=H/\ln\left(P_{i}\right),$$

where *S* is the number of species present in the community; *P_i_* is the relative advantage of species *i*.

(3) *β* diversity

The calculation formula for the Jaccard similarity index is as follows [52]:

(6) J=a/(a+b+c),

where *a* is the number of species that coexist in both sample plots; *b* is the number of species that appear only in the first sample plot; *c* is the number of species that appear only in the second sample plot.

#### 4.3.2. Classification Method for Community Types

The relative dominance values of species were standardized using the vegan (v.2.6.4) package. The cluster (v2.1.4) package was used to calculate the average contour width of the different groups; the optimal number of groups for the community was determined to be 104. The vegan package and the base R package were used to calculate the Euclidean distance matrix between sample plots, and fully connected clustering was performed. A phenotypic correlation analysis was performed between the clustering results and the Euclidean distance. The clustering analysis process was completed using R software 4.3.2, and the communities were named after the dominant species, based on clustering results.

#### 4.3.3. Selection and Statistical Analysis Methods of Environmental Characteristic Factors

This study selects 19 environmental variables to explore the driving factors that affect the composition and distribution pattern of spontaneous vegetation diversity in Heilongjiang Province, based on the actual situation of the research area and referring to previous studies [14,32,53] (Table 5).

The data preprocessing and calculation were completed using the vegan package, while the string plot was drawn using the circlize (v0.4.16) package, and all statistical charts were generated through the ggplot2 (v3.5.1) package. Due to the data not following a normal distribution, the Kruskal–Wallis test was chosen to examine the differences in *α* and *β* diversity of spontaneous vegetation among different urban habitat types, and the testing process was completed using the rstatix (v0.7.2) package. The Mantel test of the vegan package was used to calculate the Spearman correlation between the Patrick richness index of spontaneous vegetation and their different species traits and various environmental characteristic factors. All of the above analyses were conducted using R 4.3.2 software. Using species matrix and environmental feature factor data for detrended correspondence analysis (DCA), the length of the DCA1 axis is greater than 3. Subsequently, canonical correspondence analysis (CCA) was applied to analyze the relationship between the dominant species in spontaneous vegetation communities and the environmental variables. The analysis was performed using Canoco 5 software.

## 5. Conclusions

Through field investigations of urban spontaneous vegetation across different climate zones of Heilongjiang Province, it is observed that Heilongjiang Province has abundant spontaneous vegetation resources, which are influenced by different environmental factors and which have formed diverse spontaneous vegetation communities in different habitat types. Due to the complexity and heterogeneity of urban habitat types, factors affecting the diversity and community distribution of spontaneous vegetation require additional investigation in different regions and urban environments. Applying ecological indicators and methods to the study of plant communities in human-disturbed habitats also requires further verification. In addition to its important role in conserving and restoring biodiversity, spontaneous vegetation also displays enormous potential for creating new vegetation community types and wild landscapes adapted to urban biomes. To provide practical guidance for constructing low-cost, low-maintenance urban landscapes that support biodiversity and sustainability, it is essential to closely monitor invasive plants based on their habitat preferences and characteristics when establishing spontaneous vegetation communities.

## Figures and Tables

**Table 1:** Composition of life forms of urban spontaneous plants in Heilongjiang Province.

Life Form	Harbin		Mudanjiang		Yichun		Tailai		Mohe		Total	
	Species	Percentage (%)	Species	Percentage (%)	Species	Percentage (%)	Species	Percentage (%)	Species	Percentage (%)	Species	Percentage (%)
Annual or Biennial Herbaceous Plants	130	36.8	105	37.5	98	32.3	66	46.8	47	28.3	196	31.0
Perennial Herbaceous Plants	140	39.6	108	38.6	142	46.9	49	34.8	93	56.0	283	45.8
Trees	33	9.3	36	12.9	29	9.6	12	8.5	14	8.4	71	11.2
Shrubs	36	10.2	26	9.3	29	9.6	12	8.5	10	6.0	65	10.3
Ferns	1	0.3	0	0.0	2	0.7	0	0.0	0	0.0	3	0.5
Vines	13	3.7	5	1.8	3	1.0	2	1.4	2	1.2	15	2.4

**Table 2:** Overview of the origins of urban spontaneous vegetation in Heilongjiang Province.

City	Native Plants		Non-Native Plants		Invasive Plants		
	Species	Percentage (%)	Species	Percentage (%)	Species	Percentage (of Total Species) (%)	Percentage (of Nonnative Plants) (%)
Harbin	243	68.8	110	31.2	43	12.2	39.1
Mudanjiang	177	63.2	103	36.8	42	15.0	40.1
Yichun	219	72.3	84	27.7	32	10.6	38.1
Mohe	127	76.5	39	23.5	18	10.8	46.2
Tailai	94	66.7	47	33.3	19	13.5	40.4
Total	429	67.8	204	32.2	63	10.0	30.9

**Table 3:** Distribution area types of urban spontaneous vegetation families in Heilongjiang Province.

Nature	Geographic Component	Harbin Number of Families/ Percentage (%)	Mudanjiang Number of Families/ Percentage (%)	Yichun Number of Families/ Percentage (%)	Mohe Number of Families/ Percentage (%)	Tailai Number of Families/ Percentage (%)
Cosmopolitan	1. Widespread (Cosmopolitan)	33/100	27/100	31/100	20/100	19/100
Tropical	2. Pantropical (Tropically Widespread)	16/20.8	15/24.2	12/16.2	7/17.1	10/25
	2S. Pantropical, Primarily in the Southern Hemisphere	1/1.3	1/1.6	1/1.5	1/2.4	1/2.5
	3. Disjunct Distribution between East Asia (Tropical and Subtropical) and Tropical South America	2/2.6	-	1/1.5	-	-
	(3i). Non-Tropical Central and South America (Along the Andes)	1/1.3	1/1.6	-	-	-
	4. Old World Tropics	1/1.3	1/1.6	1/1.5	1/2.4	1/2.5
Subtotal		23/52.3	19/54.3	16/44.4	10/47.6	13/61.9
Temperate	8. Northern Temperate Zone	5/6.5	6/9.7	8/11.8	5/12.2	4/10.0
	8-4. Disjunct Distribution between Northern and Southern Temperate Zones	10/13.0	8/12.9	9/13.2	3/7.3	3/7.5
	9. Disjunct Distribution between East Asia and North America	3/3.9	1/1.6	2/2.9	2/4.9	1/2.5
	10. Old World Temperate Zone	1/1.3	-	-	-	-
	(12) s.s. Mediterranean Distribution	1/1.3	-	-	-	-
	12-3. Disjunct Distribution from the Mediterranean to Temperate-Tropical Asia, Oceania, and/or Southern North America to South America	-	-	-	1/2.4	-
	12-1. Disjunct Distribution from the Mediterranean to Central Asia and Southern Africa and/or Oceania	1/1.3	1/1.6	1/1.5	-	-
Subtotal		21/47.7	16/45.7	20/55.6	11/53.4	8/38.1
Total		44/100	35/100	36/100	21/100	21/100

**Table 4:** Recommendations for the application of indigenous plants and control of invasive species in urban areas of Heilongjiang Province.

Urban Habitat Types	Recommended Species with Application Potential	Recommended Invasive Species for Monitoring
Artificial turf habitat	*Argentina anserina, Arundinella hirta, Deyeuxia pyramidalis, Erysimum cheiranthoides, Trifolium lupinaster, Galeopsis bifida, Bromus inermis, Pseudolysimachion longifolium, Ixeris chinensis, Inula japonica, Lysimachia barystachys, Carex neurocarpa, Erigeron acris, Thlaspi arvense, Mentha sachalinensis, Potentilla kleiniana, Spiranthes sinensis, Pulsatilla dahurica, Elymus excelsus, Saussurea pulchella, Thalictrum simplex, Elymus ciliaris, Linaria vulgaris subsp. chinensis, Scutellaria baicalensis, Eruca vesicaria subsp. sativa, Gueldenstaedtia verna, Medicago lupulina, Hypochaeris ciliata, Zoysia japonica, Viola mandshurica, Digitaria sanguinalis, Potentilla fragarioides, Crepidiastrum sonchifolium, Elymus dahuricus, Eragrostis ferruginea, Chelidonium majus, Lepidium apetalum, Agrimonia pilosa, Mentha canadensis, Potentilla chinensis, Potentilla freyniana, Geranium wilfordii, Geranium sibiricum, Potentilla supina, Rumex acetosa, Taraxacum mongolicum, Oxalis corniculata, Portulaca oleracea, Setaria viridis, Setaria faberi, Setaria pumila, Viola philippica, Plantago asiatica, Plantago major, Plantago media, Sanguisorba tenuifolia var. alba, Arthraxon hispidus, Hieracium umbellatum, Atractylodes lancea, Circaea cordata, Lactuca indica, Euphorbia humifusa, Rorippa globosa, Agastache rugosa, Lespedeza davurica, Allium macrostemon*	*Amaranthus blitum, Galinsoga parviflora, Capsella bursa-pastoris, Ipomoea purpurea, Chloris virgata, Erigeron canadensis, Cosmos bipinnatus, Sonchus oleraceus, Abutilon theophrasti, Chenopodiastrum hybridum, Parthenocissus quinquefolia, Amaranthus tricolor, Chenopodium ficifolium, Physalis philadelphica, Trifolium repens, Amaranthus retroflexus, Veronica persica, Helianthus tuberosus, Stellaria aquatica, Hibiscus trionum, Cannabis sativa, Oxybasis glauca, Hordeum jubatum, Trifolium pratense, Ipomoea nil, Senecio vulgaris, Lolium perenne, Zephyranthes candida, Erigeron annuus, Ricinus communis, Datura stramonium, Ambrosia trifida, Zinnia elegans, Cyclachaena xanthiifolia, Saponaria officinalis, Amaranthus caudatus, Abelmoschus esculentus, Oenothera biennis, Phytolacca americana, Lepidium virginicum, Crepis tectorum, Taraxacum officinale, Festuca arundinacea, Cyperus rotundus, Iris pseudacorus, Cichorium intybus, Glebionis coronaria, Ambrosia artemisiifolia, Symphytum officinale, Galinsoga quadriradiata, Dysphania ambrosioides, Ranunculus muricatus, Solanum americanum, Sonchus asper, Cyclospermum leptophyllum, Portulaca grandiflora, Rudbeckia laciniata, Centaurea cyanus, Bromus catharticus, Oenothera glazioviana.*
Forest crevice habitat	*Solanum nigrum, Aster tataricus, Picris japonica, Aquilegia viridiflora, Persicaria senticosa, Androsace filiformis, Potentilla flagellaris, Deyeuxia pyramidalis, Galium boreale, Bupleurum longiradiatum, Galium verum, Pyrola asarifolia subsp. incarnata, Saussurea pectinata, Euphorbia esula, Takhtajaniantha austriaca, Anemone dichotoma, Artemisia igniaria, Hylotelephium pallescens, Silene repens, Thalictrum aquilegiifolium var. sibiricum, Filipendula digitata, Impatiens noli-tangere, Polygonatum stenophyllum, Pleurospermum uralense, Lysimachia davurica, Viola tenuicornis, Lactuca triangulata, Viola mongolica, Atractylodes lancea, Viola phalacrocarpa, Impatiens furcillata, Pinellia ternata, Isodon excisus, Moehringia lateriflora, Sahashia stricta, Woodsia ilvensis, Viola collina, Astragalus membranaceus, Potentilla cryptotaeniae, Potentilla kleiniana, Carex siderosticta, Viola selkirkii, Geranium maximowiczii, Heracleum moellendorffii, Spiranthes sinensis, Stellaria palustris, Trigonotis radicans, Saussurea japonica, Saussurea pulchella, Elymus ciliaris, Picris hieracioides, Lespedeza bicolor, Artemisia japonica, Conioselinum smithii, Persicaria dissitiflora, Sanguisorba tenuifolia var. alba, Adoxa moschatellina, Leibnitzia anandria, Dryopteris amurensis, Athyrium brevifrons, Clematis fusca, Ranunculus japonicus, Aster pekinensis, Carex leiorhyncha, Catolobus pendulus, Pilea pumila, Rubia sylvatica, Potentilla fragarioides, Potentilla longifolia, Lactuca indica, Glycine soja, Glechoma longituba, Chelidonium majus, Centipeda minima, Potentilla chinensis, Agrimonia pilosa, Stellaria radians, Viola prionantha, Viola acuminata, Geranium wilfordii, Geranium sibiricum, Mazus pumilus, Acalypha australis, Viola philippica, Oxalis corniculata, Polygonum aviculare, Stellaria media, Inula britannica, Schisandra chinensis, Kummerowia striata, Aster hispidus, Commelina communis*	
Grassland crevice habitat	*Achillea alpina, Sanguisorba officinalis, Persicaria longiseta, Lespedeza davurica, Chamerion angustifolium, Picris japonica, Arthraxon hispidus, Deyeuxia pyramidalis, Galium boreale, Artemisia gmelinii, Artemisia igniaria, Galeopsis bifida, Campanula punctata, Campanula glomerata, Lysimachia davurica, Lysimachia barystachys, Melampyrum roseum, Viola collina, Astragalus membranaceus, Spiranthes sinensis, Trigonotis radicans, Saussurea japonica, Linaria vulgaris subsp. chinensis, Lespedeza juncea, Lespedeza bicolor, Artemisia japonica, Gueldenstaedtia verna, Medicago lupulina, Zoysia japonica, Viola acuminata, Aster pekinensis, Aster lautureanus, Menispermum dauricum, Potentilla fragarioides, Potentilla longifolia, Lactuca indica, Aster hispidus, Glycine soja, Clematis brevicaudata, Commelina communis, Agrimonia pilosa, Stellaria radians, Viola prionantha, Potentilla chinensis, Potentilla freyniana, Geranium wilfordii, Geranium sibiricum, Potentilla supina, Polygonum aviculare, Cynanchum rostellatum, Viola philippica, Perilla frutescens, Allium macrostemon, Rubus idaeus, Thalictrum aquilegiifolium var. sibiricum, Atractylodes lancea, Potentilla kleiniana, Oxalis corniculata.*	
Gravel-type abandoned land and barren soil abandoned land habitat	*Eragrostis pilosa, Eragrostis minor, Arundinella hirta, Artemisia gmelinii, Calamagrostis macrolepis, Ixeris chinensis, Erigeron acris, Sisymbrium officinale, Cynanchum thesioides, Medicago ruthenica, Inula linariifolia, Elymus ciliaris, Lespedeza juncea, Bidens tripartita, Salsola collina, Medicago lupulina, Persicaria lapathifolia, Bassia scoparia, Persicaria orientalis, Artemisia sieversiana, Digitaria sanguinalis, Elymus kamoji, Thladiantha dubia, Amethystea caerulea, Crepidiastrum sonchifolium, Elymus dahuricus, Aster hispidus, Leonurus sibiricus, Digitaria ischaemum, Plantago depressa, Trigonotis peduncularis, Artemisia annua, Plantago major, Bidens parviflora, Euphorbia humifusa, Potentilla chinensis, Potentilla freyniana, Calystegia hederacea, Taraxacum mongolicum, Viola philippica, Setaria viridis, Setaria faberi, Setaria pumila, Cynanchum rostellatum, Portulaca oleracea, Arthraxon hispidus, Persicaria longiseta, Potentilla kleiniana, Leibnitzia anandria, Lespedeza davurica, Allium macrostemon, Erysimum cheiranthoides, Bromus inermis, Pulsatilla dahurica, Saussurea pulchella, Scutellaria baicalensis, Eruca vesicaria subsp. sativa, Zoysia japonica, Potentilla supina, Trifolium lupinaster, Viola mandshurica.*	

**Table 5:** Selection of environmental characteristic factors and their represented meanings.

Name of Environmental Characteristic Factor	Data Source and Calculation Method	Significance
Patch area (Area)	Generated through geometric calculations using ArcGIS 10.8 software.	Used to reflect the edge effects around patches.
Perimeter–Area Ratio of Patches (P/A)	P/A	
Patch Landscape Shape Index (LSI)	$P/2\sqrt{\pi \times A}$	
Relative Richness of Cultivated Plants in Patches (ICNCP)	P/S	Used to evaluate the impact of vegetation structure within patches on the diversity of indigenous plants.
Percentage of Woody Cover in Patches (PC)	Field survey records	
Average Distance from Patch to Urban Edge (BD)	Obtained using the Nearest Neighbor Analysis tool in ArcGIS 10.8 software.	Characterizes the degree of urbanization and the distance between patches and seed sources.
Distance between Neighboring Patches (NN)		Used to assess species dispersal resistance.
Impervious Surface within 50/100/300/500 m Buffer of Patches (Sealed_50/100/300/500_)	Retrieved from the ESA website, the land cover dataset is used to calculate the ratio of urban construction land to total land area within the patch buffer using ArcGIS 10.8 software.	
Building Volume within 50/100/300/500 m Buffer of Patches (BV_50/100/300/500_)	Retrieved from the ESA website, the building height data is used to calculate the product of building height and building area within the patch buffer using ArcGIS 10.8 software.	Analyzes urbanization in the vertical space of the city.
Population Density within 50/100/300/500 m Buffer of Patches (POP_50/100/300/500_)	Retrieved from the Fitshare website, the population dataset is used to calculate the ratio of population number to buffer area within the patch buffer using ArcGIS 10.8 software.	Used to evaluate the intensity of human activity disturbance.

## Data Availability

The original data presented in the study are openly available in Zenodo at 10.5281/zenodo.13895355.

## Supplementary Materials

The following supporting information can be downloaded at: https://www.mdpi.com/article/10.3390/plants13212982/s1, Figure S1: Cluster analysis of spontaneous plant communities based on Jaccard similarity index; Table S1: Eigenvalues and cumulative variance for each axis in Canonical Correspondence Analysis (CCA); Table S2: Correlation matrix between axes and environmental variables in Canonical Correspondence Analysis (CCA); Table S3: Explanatory power, contribution rate, and significance statistics of environmental factors in Canonical Correspondence Analysis (CCA).
