# Supplementary material for: Distribution Patterns of Urban Spontaneous Vegetation Diversity and Their Response to Habitat Heterogeneity: A Case Study of Five Cities in Heilongjiang Province, China
Source: Plants (Basel). 2024 Oct 25;13(21):2982. doi: 10.3390/plants13212982 (PMC11547939; doi:10.3390/plants13212982)
Supplement: Supplementary file 1 [file plants-13-02982-s001.zip › plants-3237212-supplementary.pdf]

Figure S1: Cluster analysis of spontaneous plant communities based on Jaccard similarity index;

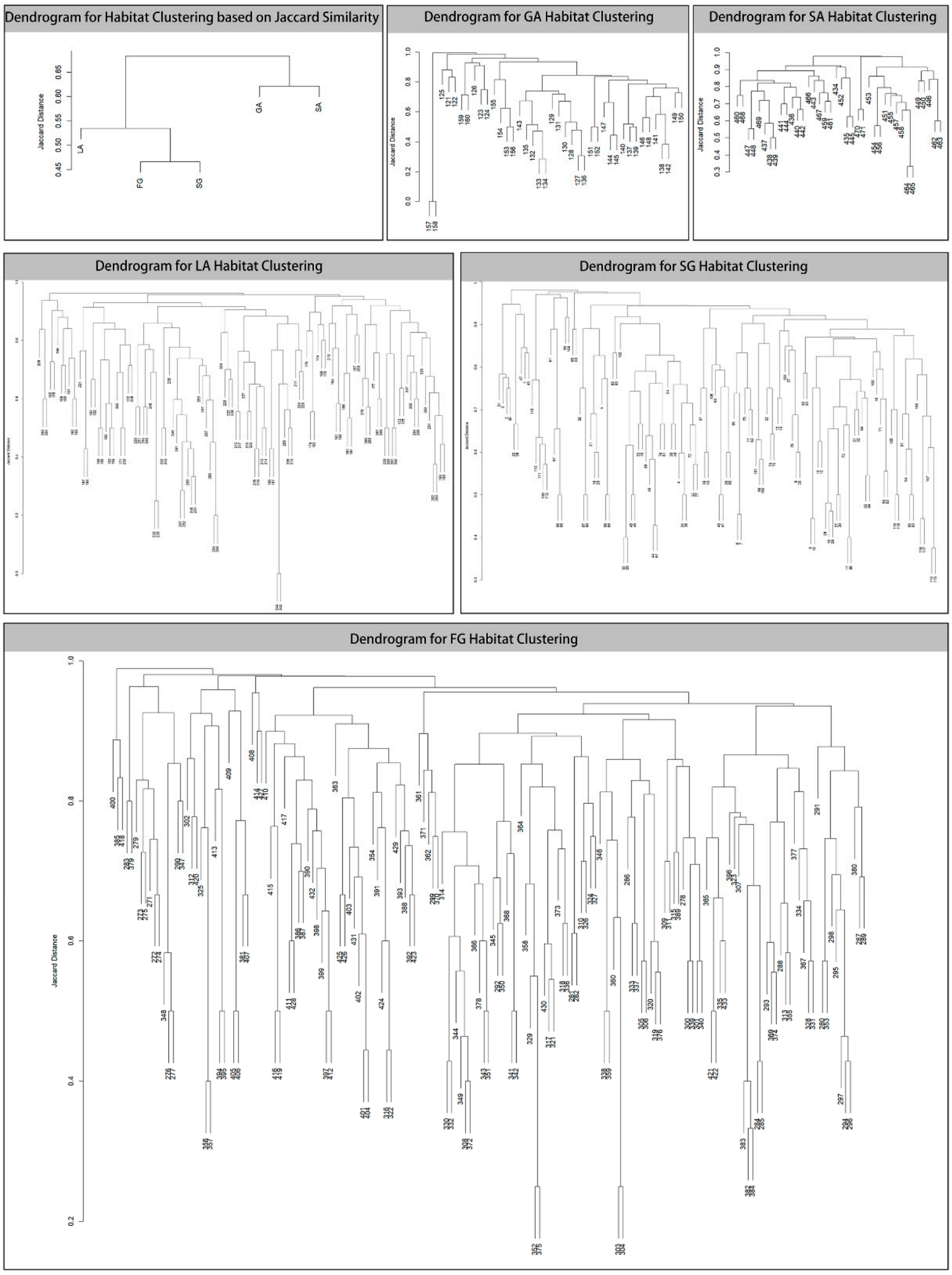

Table S1: Eigenvalues and cumulative variance for each axis in Canonical Correspondence Analysis (CCA);

Axis	Eigenvalue	response data (%)	fitted response data (%)
1	0.51418	1.1	14.7
2	0.32368	1.8	24
3	0.26870	2.4	31.7
4	0.25857	2.9	39.2

Table S2: Correlation matrix between axes and environmental variables in Canonical Correspondence Analysis (CCA);

Variable	Resp Ax1	Resp Ax2	Resp Ax3	Resp Ax4
BD	-0.4668	-0.0632	-0.0116	0.3189
NN	0.5636	0.0101	0.2969	0.2236
PC	-0.4745	0.1446	0.1515	0.1271
ICNCP	-0.2229	0.1834	-0.1622	0.2355
Area	-0.4223	0.2077	0.2193	-0.037
P/A	0.1882	-0.0848	-0.1739	0.1883
LSI	-0.4662	0.1005	0.1187	-0.1572
Sealed50	0.3676	-0.1177	-0.0427	0.4152
Sealed100	0.3115	-0.1302	-0.0704	0.4318
Sealed300	0.1533	-0.2768	-0.0862	0.4321
Sealed500	0.0751	-0.2724	-0.087	0.4579
BV50	-0.3364	0.148	0.2092	0.0848
BV100	-0.3152	0.138	0.1891	0.1042
BV300	-0.3321	0.0088	0.1191	0.1373
BV500	-0.3845	-0.0613	0.0626	0.2132
POP50	-0.1932	-0.0751	0.0071	0.3675
POP100	-0.232	-0.0808	0.0144	0.3585
POP300	-0.3282	-0.0883	-0.0431	0.3645

POP500	-0.4002	-0.12	-0.0236	0.3771

Table S3: Explanatory power, contribution rate, and significance statistics of environmental factors in Canonical Correspondence Analysis (CCA).

**Name**	Explains %	Contribution %	pseudo-F	P
NN	0.8	10.1	3.6	0.001
PC	0.6	7.5	2.7	0.001
BD	0.5	6.8	2.4	0.001
Area	0.5	6.3	2.3	0.001
sealed500	0.5	6.1	2.2	0.001
BV100	0.4	5.7	2.1	0.001
ICNCP	0.4	5.4	1.9	0.001
BV300	0.4	5.3	1.9	0.001
BV500	0.4	4.8	1.8	0.001
sealed300	0.4	5.3	1.9	0.001
sealed100	0.4	5	1.8	0.001
P/A	0.3	4.2	1.5	0.016
POP300	0.3	4.2	1.5	0.007
POP500	0.3	4.1	1.5	0.001
POP100	0.3	4.4	1.6	0.003
sealed50	0.3	4.2	1.5	0.095v
LSI	0.3	3.9	1.4	0.021
POP50	0.3	3.4	1.2	0.09
BV50	0.2	3.3	1.2	0.112
